# Longitudinal measurement of cortisol in association with mental health and experience of domestic violence and abuse: study protocol

**DOI:** 10.1186/1471-244X-13-188

**Published:** 2013-07-13

**Authors:** Natalia V Lokhmatkina, Gene Feder, Sarah Blake, Richard Morris, Victoria Powers, Stafford Lightman

**Affiliations:** 1 School of Clinical Sciences, University of Bristol, Dorothy Hodgkin Building, Whitson Street, Bristol, UK; 2 Centre for Academic Primary Care, University of Bristol, Canynge Hall, 39 Whatley Road, Bristol, UK; 3 Primary Care and Population Health Department, University College London, Royal Free Campus, Rowland Hill Street, London, UK; 4 Department of Clinical Biochemistry, Bristol Royal Infirmary, Marlborough Street, Bristol, UK

**Keywords:** Domestic violence, Partner abuse, Spousal abuse, Battered women, Abused women, Cortisol, Mental health, Depression, Anxiety, Posttraumatic stress disorder

## Abstract

**Background:**

Domestic violence and abuse is threatening behavior, violence/abuse used by one person to control the other within an intimate or family-type relationship. Women experience more severe physical and sexual domestic violence and abuse and more mental health consequences than men. The current study aims at exploring of the role of hypothalamic-pituitary-adrenocortical axis activity in abuse impact on women's mental health. Study objectives: 1) To evaluate diurnal cortisol slope, cortisol awakening response, and the mean cortisol concentration in women with a current or recent experience of abuse; 2) To estimate whether cortisol secretion is associated with type, severity, duration and cessation of abuse; 3) To investigate whether cortisol acts as mediator between abuse and mental health condition; 4) To examine whether there is any distinction in cortisol levels between those women exposed to both childhood abuse and domestic violence and abuse and those experienced only the latter. 4) To explore whether cortisol secretion differs between women living in refuge and those still living in the community.

**Methods/Design:**

To meet study objectives 128 women will be recruited in a domestic violence agency and local communities. Baseline and 3-month follow-up measures will be taken over 6 months after recruitment. Each assessment will include: (1) standardized self-administered questionnaires to evaluate socio-demographics, experience of violence and abuse, mental and physical health; (2) weight and height measurement; (3) self-completion of wakening, post-wakening and evening saliva samples. Saliva will be analysed for cortisol and cortisone using Ultra performance liquid chromatography – tandem mass spectrometry.

We will compare diurnal cortisol parameters between non-abused controls and abuse survivors with and without mental health conditions. First following descriptive statistics for all the cortisol and mental health outcomes, relationships between them will be investigated using appropriate regression models. Second, these techniques will be used to investigate the extent to which cortisol measures act as potential mediators between type, severity, duration of abuse and mental disorders.

**Discussion:**

Results of the study will increase our understanding of the pathophysiological mechanisms of abuse-related mental health disorders in women and inform researchers and practitioners on the possibility of using salivary cortisol as a biological marker for prognosis, diagnosis, and treatment evaluation among abuse survivors.

**Trial registration:**

ClinicalTrials.gov registration NCT01632553

## Background

Domestic violence and abuse (DVA) is threatening behavior, violence or abuse (psychological, physical, sexual, financial or emotional) between adults who are or have been intimate partners or family members, regardless of gender and sexuality. DVA can be better understood as a chronic syndrome characterized not only by episodes of physical violence but also by the emotional and psychological abuse the perpetrators use to maintain control over their partners [1]. Life time prevalence of DVA is 28% for women and 18% for men, although severity and consequences of abuse are less for men [2]. Over and above damage to physical and reproductive health DVA has long-term detrimental effects on mental health for women consulting in primary care [3,4]. High prevalence of lifetime and past year DVA is reported in psychiatric female patients [5]. A meta-analysis of (mostly) North American studies measuring the relationship between DVA and mental disorders reported increased risk for depression, anxiety, psychosomatic disorders, posttraumatic stress disorder (PTSD), alcohol abuse, and suicidal behavior [6]. Compared to non-abused women, female victims of DVA visit their general practitioners more frequently. They are also more often referred to additional diagnostics and mental health care, as well as prescribed antidepressants [7]. Kernic et al. [8] have established that cessation of DVA among survivors is associated with decreased prevalence of depression; whereas Anderson and Sounders [9] have found that some women out of the abusive relationship may have greater psychological difficulties than those who are still in it. Blasco et al. [10] concluded that the pattern of mental health recovery depends on the type of DVA that the women had been exposed to.

In addition, the mechanisms through which DVA causes mental and physical conditions are very poorly studied. One mechanism that may contribute to stress-related disorders is the hypothalamic-pituitary-adrenocortical (HPA) axis, which produces cortisol [11-13]. Cortisol activity in healthy children and adults follows a consistent diurnal pattern with peak levels of output observed within the first 30–40 min after awakening followed by a progressive reduction throughout the morning and afternoon with a nadir around midnight [14]. Cortisol levels naturally increase in response to exposure to acute stress and helps organisms cope with a short term homeostatic challenges by modification of metabolic and cognitive function [15]. Chronic stress has the capacity to increase or decrease HPA activity, and the resulting pattern depends, both on features of the stressor and the person facing it. Female survivors describe DVA as traumatic uncontrollable stress that poses a threat to their physical and social self, and is associated with feelings of shame [16]. There are very few studies focused on assessing physiologic correlates of chronic stress in abused women.

Salivary cortisol has been successfully used as a biological marker of HPA axis activity in epidemiological studies [17,18] including samples of women exposed to DVA [19-21]. Diurnal cortisol slope, cortisol awakening response (CAR), and area under the daytime cortisol curve have been found the most robustly related to psychosocial phenomena and to health outcomes [22]. Systematic review of the endocrine sequelae in chronically stressed but non psychiatric caregivers for family members reveals four studies which reported no difference, five studies reporting increased cortisol output, and four studies reporting hypo- secretion of cortisol compared to controls [23]. Powell et al. [20] have found out that women chronically stressed by undergoing a divorce or separation displayed elevated evening salivary cortisol compared to no stressed women in stable marriages. Moreover, it was the chronic stress rather than depression or acute stress that accounted for the elevation of evening salivary cortisol. Systematic review and meta-analysis of cortisol and posttraumatic stress disorder revealed significantly lower cortisol levels in people with PTSD due to sexual or physical abuse than controls. Interestingly, no difference in cortisol levels was found between controls and people with PTSD due to other types of trauma [24]. In contrast, anxiety disorder was associated with higher awakening cortisol levels [25]. Meta-analysis of studies meeting methodological standards showed that depression was associated with small-to-moderate elevation in cortisol output [26].

Long-term studies are absent in the field of pathophysiology of DVA [22]. The results of existing cross-sectional and short-term longitudinal studies testing the impact of childhood abuse and DVA on women’s HPA axis functioning are contradictory (see Additional file 1).

It is evident that most investigators have reported different patterns of alterations in diurnal cortisol output in women exposed to childhood abuse (CHA) and DVA. However there are researchers who have concluded that cortisol is a marker of abuse exposure rather than mental health diagnosis [27,28]; others stated that alteration in cortisol output is a correlate of abuse-related mental disorder [29,30]. Currently, it is known that CHA influences the psychobiology of cortisol function and that there is a development from hyper secretion of cortisol to hypo secretion by early adulthood [14]. It is also known that both physical and psychological DVA have significant impacts on the neuroendocrine system of women [27]. At the same time it is not clear whether currently and recently abused women, who have abnormal diurnal cortisol profiles, demonstrate poorer mental health outcomes and how this may correlate with type, severity, duration and cessation of abuse. There is a hypothesis that DVA survivors may be characterized by alterations in HPA axis activity [31] and that further longitudinal studies are needed to identify specific stress system disturbances in this group [2,32]. The current research study aims to investigate the role of the HPA axis on the impact of DVA on women’s mental health. This study tests the hypothesis that compared to non-abused controls all abused women have altered diurnal rhythm in cortisol secretion and that the pattern of this alteration is predicted by abuse characteristics, such as its type, severity, duration, and cessation.

### Study objectives

1. To evaluate the key diurnal cortisol parameters (diurnal cortisol variation, CAR, and mean cortisol concentration) in women with a current or recent experience of DVA

2. To estimate whether cortisol parameters are associated with type, severity, duration and cessation of abuse

3. To investigate whether cortisol outcomes act as a mediator between the features of abuse and mental health state

4. To examine whether there is any distinction in cortisol levels between those women exposed to both CHA and DVA and those experienced only the latter

5. To explore whether cortisol secretion differs between women, living in a domestic violence refuge and those still living in the community (after adjustment for confounding effects of abuse severity and continuing contact with abuser).

## Methods/Design

The study was approved by a Research Ethics Committee established by the NHS Health Research Authority (NRES Committee South West – Frenchay, REC reference 12/SW/0162 of 25 June 2012). This is a 6-month, prospective, observational study based on a convenience sample. We will recruit index participants from the pool of women referred to specialized domestic violence agency SURVIVE South Gloucestershire and Bristol for community outreach support or refuge accommodation by other agencies or self-referred. The SURVIVE agency is a charitable organization which provides a range of services to women who have experienced DVA, including three refuges for women with children, outreach support, community-based Freedom Program groups, Independent Domestic Violence Advisers, support phone line, and Information Sessions in South Gloucestershire, and Bristol UK.

Inclusion criteria for participants contain age ≥ 18 y.o. Exclusion criteria include inability to read English, current use of steroid-based medications, pregnancy, presence of pituitary and/or adrenal gland disorder, and symptomatic psychotic illness. No participant will be excluded on the basis of their disability, ethnicity, religion or sexual orientation.

### Recruitment

Potential index participants will be identified by written invitation letters with invitation slip attached offered by researcher or agency staff to every woman referred or self-referred to domestic violence agency SURVIVE. Woman willing to be approached to discuss potential participation in the study can either send her contact details to the researcher by phone or e-mail, or put down her contact details on the invitation slip, tear it off and return to the researcher or agency staff who has given the invitation to her.

The researcher will collect contact details sent by phone, e-mail, and completed invitation slips and phone all women willing to be approach to hear more about the study. When initial telephone contact has been made with a woman, the researcher will assess her eligibility and arrange a face-to-face meeting with all women interested and eligible to take part, meeting the woman at a safe and convenient place. As part of the first face-to-face meeting, the researcher will give a detailed participant information sheet to the woman, explaining each point and answer any questions that the woman may have prior to seeking her written informed consent.

The researcher will ask every woman already consented to participate in the study to suggest up to three female friends or family members of the same age group who are aware of her current circumstances to be her potential controls. The researcher will emphasize that suggesting friend controls is completely optional. If a woman does not want to recommend friend controls it will not affect her participation in the study nor the services she receives. If a woman wishes to suggest friends as potential controls the researcher will ask her to pass ‘An invitation to the CEASE study’ to those people. ‘An invitation to the CEASE study’ and all documentation for friend controls present the study as biological research on the impact of family stress on women's health and wellbeing and do not mention SURVIVE agency. Although it does state that the research is about DVA, the researcher will ask the index participant only to pass the invitation to confidantes who know that she has experienced abuse. A friend control willing to discuss potential participation in the study will send her contact details to the researcher by post, phone, text, or e-mail. The researcher will contact potential friend controls by phone to give brief information about the study, assess their eligibility and arrange a face-to-face meeting at a safe and convenient place. As part of the first face-to-face meeting, the researcher will give a detailed participant information sheet to the friend control, explaining each point and answer any questions that the woman may have prior to seeking her written informed consent. In parallel with recruitment of friend controls through index research participants we will approach potential controls in local communities through invitation letters displayed in public places (e.g. post office, library, supermarket, community centre) in areas surrounding SURVIVE sites in South Gloucestershire and Bristol.

### Measurements

This study will consist of one baseline and two follow-up measurements. Baseline evaluation will be carried out at the first face-to-face meeting between researcher and woman and during the following week. Women will be asked to undertake follow-up assessment at 3 and 6 months after joining the study. With regard to safety preferences participants will have a choice to receive and return follow-up questionnaire and saliva collection kit by post or at the follow-up face-to-face meeting with the researcher. Each assessment will last approximately 45 minutes and will include: (1) numerous standardized self-administered questionnaires to assess socio-demographics, mental and physical health, (2) weight and height measurement, and (3) self-completion of three saliva samples. The questionnaire and saliva samples will be completed by the participant at a place that is safe and convenient to her and returned by post or via collection by the researcher. Research plan and measurements are summarized in Additional file 2.

Socio-demographic information will be collected through use of a standardized self-report questionnaire and include age, ethnicity, children, qualifications, employment status, total annual income and relationship to perpetrator.

Exposure to abuse through the lifespan will be measured using two psychological instruments. First, cases of childhood abuse and neglect will be detected using the Childhood Trauma Questionnaire (CTQ). CTQ is a 28-item inventory that provides brief, reliable and valid screening for histories of abuse and neglect. The CTQ inquires about five types of maltreatment – emotional, physical, and sexual abuse, and emotional and physical neglect – with five items representing each type. Participants respond to a series of statements about childhood events, which are endorsed on a 5-point frequency scale (0 = “never true”, 4 = “very often true”). Item scores are then summed to produce scores that quantify the severity of maltreatment in each area and that can be compared to clinical data. Cut-off scores for detecting likely cases of abuse and neglect are also provided. The CTQ was validated with data from over 2,000 respondents, including both clinical and non referred groups [33]. Second, exposure to DVA from intimate partner and other family members will be measured using the Composite Abuse Scale (CAS). The CAS is a widely used self-report of behaviors that women describe as abusive by their partners [34]. It is an easily administered measure that provides standardized sub scale scores on four dimensions of DVA. It consists of 30 items presented in a six point format requiring respondents to answer “never”, “only once”, “several times”, “monthly”, “weekly” or “daily” in a twelve month period. The Severe Combined Abuse sub scale has 8 items that represent severe physical abuse items, all sexual abuse items, and physical isolation aspects of emotional abuse. The Emotional Abuse subscale has 11 items that include verbal, psychological, dominance and social isolation abuse items. The Physical Abuse subscale has 7 of the less severe physical abuse items and the Harassment subscale has 4 items that are about actual harassment. CAS is scored by summating the frequency scores of the 30 items. The 0–5 for each item thus gives a possible score for each sub scale. Cut-off scores to exclude women labeled abused incorrectly are provided. The CAS can give not only prevalence figures, but also associations with other physical and emotional co-morbidities. It has been used in specialized domestic violence agencies, general practice, antenatal clinics, emergency departments, and drug and alcohol clinics. The CAS has demonstrated good acceptability and reliability, with some evidence of criterion and external validity. To measure DVA duration an item is added to the CAS asking participants the length (“one occasion”, “up to 6 months”, “up to 1 year”, “up to 3 years”, “up to 5 years”, and “more than 5 years”) of abusive relationship.

Levels of stress and mental health of the participants will be evaluated using five psychological instruments for detecting the most common mental disorders alongside with standardized questionnaire on use of alcohol and drugs. First, the Generalized Anxiety Disorder Scale (GAD-7) will be used to screen on probable cases of generalized anxiety disorder and to assess symptom severity. The GAD-7 is based on the most prominent diagnostic features of the DSM-IV diagnostic criteria for generalized anxiety disorder and has excellent reliability, as well as criterion, construct, factorial, and procedural validity. The 7 items assess the frequency of core symptoms of generalized anxiety disorder within the past two weeks. Items are rated on a 4-point frequency scale (0 = “not at all”, 3 = “nearly every day”) with a total score ranging from 0 to 21. A score up to 4 indicates the absence of generalized anxiety disorder, scores of 5–9 represent mild, scores of 10–14 represent moderate and scores of 15 and higher represent severe anxiety symptoms levels [35].

Second, Patient Health Questionnaire Depression Scale (PHQ-9) will be used to diagnose major depression and assess symptom severity. The PHQ-9 is based on the DSM-IV diagnostic criteria for major depressive disorder and has excellent reliability, as well as criterion, construct, factorial and procedural validity. The 9 items assess the frequency of depressive symptoms within the past two weeks. Items are scored on a 4-point frequency scale from 0 = “not at all” to 3 – “nearly every day” with a total score ranging from 0 to 27. A cut-off point of >9 is recommended for the screening of any depressive disorder. A score up to 4 indicates the absence of depression, scores of 5–9 represent mild, scores of 10–14 represent moderate and scores of 15 and higher represent severe depression [35,36].

Third, PTSD will be diagnosed using the PTSD Symptom Scale: Self-Reported version (PSS-SR). The PSS-SR is a 17-item measure that assesses the 17 DSM-IV symptoms of PTSD. The severity over the last two weeks of each item on the PSS-SR is rated using a 4-point scale (0 = “not at all”, 4 = “every day”). The total score is calculated as the sum of severity ratings for the 17 items. The total score > 13 indicates on likelihood of PTSD. The PSS-SR has satisfactory internal consistency, high test-retest reliability, and good concurrent validity [37].

Fourth, the Sheehan Disability Scale (SDS) will be used to assess functional impairment in three inter-related domains; work/school, social and family life. The participant rates the extent to which work/school, social life and home life or family responsibilities are impaired by her symptoms on a 10-point visual analog scale. Score 5 or greater on any of the three scales is associated with significant functional impairment. The SDS has high sensitivity and good specificity for patients with most frequent mental disorders [38].

Fifth, the Perceived Stress Scale (PSS) will be used for measuring the perception of stress [39]. This widely used 10-item psychological instrument with good internal reliability and moderate construct validity measures the degree to which situations in one’s life are appraised as stressful over the past month. It assesses the amount of stress in one’s life rather than response to a specific stress and had been used in studies of both mental and physical health. The questions in the PSS ask about feelings and thoughts during the last month. In each case, respondents are asked how often they felt a certain way. Answers are rated on a 5-point frequency scale (0 = “never”, 4 = “very often”). PSS scores are obtained by reversing responses to the four positively stated items and then summing across all scale items. Scores can range from 0 to 40, with higher scores indicating greater stress.

Finally, use of alcohol and drugs will be measured by asking questions about quantity and frequency of substance use.

Physical health of the participants will be evaluated by body mass index (BMI) and a standardized questionnaire. To calculate BMI the researcher will follow standard clinical protocol on taking standing height and weight. Height will be measured to the nearest 1 mm (KaWe person-check, Germany), weight to the nearest 0.1 kg (Seca 803 Digital Personal Scale, Germany). Physical health problems will be measured using a modification of the Miller Abuse Physical Symptom and Injury Scale (MAPSAIS). This self-reported scale lists 9 injuries and 44 symptoms and illnesses related to DVA and asks if the woman was hospitalized, visited emergency department or undertaken surgery in the past 12 months. The scale was designed specifically for measuring long-term health consequences of DVA and demonstrated good content validity and test-retest reliability [40].

Upon completion of questionnaire and anthropometry study participants will be given a saliva sample collection kit that includes a saliva collection device (Salivette tubes) with cotton pledget, an illustrated instruction sheet written in plain language explaining the procedure and providing helpful hints (such as putting the tube next to the bed for morning sample), a saliva collection diary in which participants can record experiences on the day of sample collection and a prepaid packaging addressed to the accredited laboratory compliant with current postal regulations. The participants will be asked to collect three saliva samples as soon as possible after questionnaire survey:

1. Sample 1 “bedtime” – right before getting into bed.

2. Sample 2 “awakening” – next morning as soon as the woman wakes up and is ready to get up for the day.

3. Sample 3 “post-awakening” – 30 minutes after taking awakening sample.

Saliva will be collected by either passive drooling into the tube or by chewing (for 2 minutes) the cotton pledget provided with each tube. Participants must have waited at least 30 minutes after smoking, consuming any food or drink or teeth brushing before collecting the saliva sample. Tubes will be labeled with patient identifier and returned to the laboratory by post or via collection by the researcher. Upon arrival to the laboratory tubes with saliva will be centrifuged to remove the upper saliva layer for subsequent measurement of cortisol. The primary tube including the cotton pledget with individual cells will be then discarded in routine clinical waste. The upper saliva layer will be frozen until analysis. The saliva samples will be analyzed in the Department of Clinical Biochemistry at the Bristol Royal Infirmary using Ultra performance liquid chromatography – tandem mass spectrometry (UPLC-MSMS). The analysis also simultaneously measures cortisone a metabolite of cortisol as an additional measure of circulating cortisol [41,42].

To collect data on known covariates that have been related to diurnal cortisol secretion participants will complete saliva collection diary [22]. Data collected on the day of saliva sampling include: schedule (whether weekend vs. weekday), time of awakening, lifestyle (smoking, alcohol use, caffeine, exercise/activity level, perceived stress and negative mood, recent meal, medications) and menstrual timing.

### Statistical analysis

For the sample size calculation we used findings from similar studies in comparable populations [24,43,44]. Thus, the number of participants required to detect a standardized difference in cortisol levels of 0.5 between abused women and non-abused controls with 80% power using a cut-off score for statistical significance of 0.05 is 128 (64 per group). Assuming 40% drop out rate before the study ends, around 213 eligible subjects would have to be approached. Assuming 60% response rate we will have to approach approximately 355 women, so 213 would consent to join the study, and of these 128 would be expected to complete the study.

Data will be coded and entered into research database during data collection by the two researchers responsible for recruitment. Stata software will be used for statistical analysis. All data will be checked for expected ranges, presence of outliers and abnormal values.

Sample will be analyzed by comparison of participant exposed to DVA and participants without DVA exposure on the basis of cortisol and mental health state data. Primary study outcome will be reported as diurnal cortisol variation, the degree of decline in cortisol across the day. Secondary study outcomes include CAR, size of post-awakening surge in cortisol that occurs in 30 minutes after awakening and mean salivary cortisol concentration across the day. As an additional measure of tissue cortisol the three saliva samples, awakening, post awakening, and bedtime, will also be assayed for cortisone.

Following descriptive statistics for all the cortisol and mental health outcomes, relationship between them will be investigated using appropriate linear (and, for binary outcomes, logistic) regression models. Using the CAS and CTQ data, these techniques will also be used to investigate the extent to which cortisol measures act as potential mediator between the type, duration and cessation of abuse and mental health condition for this group of women.

Missing data from women who dropped out of the study will be handled first by comparing their baseline variables with those who completed the study, and secondly using imputation techniques [45].

## Discussion

The current study: (1) evaluates levels of stress, mental health, and stress biomarker salivary cortisol for currently and recently abused women and non-abused controls, across three time periods over 6 months; (2) explores whether there are altered diurnal patterns of cortisol secretion within this sample; and (3) identifies socio-demographic, abuse and mental health predictors of diurnal cortisol parameters. The study protocol and participant documentation have been amended based on guidance from our research advisory group (five former SURVIVE users) and the pilot study. This study is methodologically strong compared to very few neuroendocrinological studies previously conducted in abused women. Firstly we use three-point saliva sampling protocol across three time periods over 6 months which enables us to obtain the key diurnal cortisol parameters prospectively – the diurnal cortisol slope, the size of awakening response, and the average cortisol exposure across the day. Secondly we shall collect data on a number covariates related to diurnal cortisol secretion in order to control neuroendocrine variables for potential confounding. Thirdly UPLC-MSMS will be used to measure salivary cortisol. UPLC-MSMS is a superior technique to traditional immunoassay steroid methods, with improved sensitivity and specificity which is able to measure both salivary cortisol and cortisone (cortisol metabolite) in the samples simultaneously. Fourthly to measure stress, mental health and abuse we use international validated questionnaires. Finally our targeted sample size is larger than those reported in earlier studies in women exposed to DVA which allows performance of subgroup analysis stratified by mental health status, and characteristics of DVA.

This study should add significantly to our understanding of the physiological mechanisms of DVA and explain causal pathway between abuse and mental health consequences in female survivors. It will improve understanding of neuroendocrine markers associated with abuse and mental health status, potentially leading to studies of prognosis and more targeted management of women who have experienced DVA and its sequelae. Ultimately it might be possible to better target intervention for women experiencing DVA with and without specific mental health sequelae in relation to activity of HPA axis. The current study represents a step on this direction.

## Abbreviations

BMI: Body mass index; CAR: Cortisol awakening response; CAS: Composite abuse scale; CTQ: Childhood trauma questionnaire; DD: Dissociative disorder; DSM – IV: Diagnostic and statistical manual of mental disorders fourth edition; DVA: Domestic violence and abuse; GAD: Generalized anxiety disorder; HPA: Hypothalamic-pituitary-adrenocortical; IPV: Intimate partner violence; MAPSAIS: Miller abuse physical symptom and injury scale; MDD: Major depressive disorder; PHQ: Patient health questionnaire; PSS: Perceived stress scale; PSS-SR: Posttraumatic stress disorder symptom scale - self-reported; PTSD: Posttraumatic stress disorder; SDS: Sheehan disability scale; UPLC-MSMS: Ultra performance liquid chromatography – tandem mass spectrometry.

## Competing interests

The authors declare that they have no competing interests.

## Authors’ contributions

NL developed the concept and design of the study and wrote the protocol and manuscript. GF and SL framed the concept and design of the study and reviewed and edited the protocol and manuscript extensively. RM contributed to the statistical part and reviewed and edited the manuscript. SB participated in the design of the study and reviewed and edited the manuscript. VP contributed to the laboratory assay part of the protocol and reviewed and edited the protocol and manuscript. All authors read and approved the final manuscript.

## Pre-publication history

The pre-publication history for this paper can be accessed here:

http://www.biomedcentral.com/1471-244X/13/188/prepub

## Supplementary Material

Additional file 1 **Diurnal cortisol activity in women exposed to childhood abuse (CHA) and DVA.** PDF table summarizing methodological information about publishes studies on HPA axis activity in association with exposure to violence and abuse. Click here for file

Additional file 2 **Study plan and measurements.** PDF table summarizing CEASE study plan and measurements. Click here for file
