# Supplementary material for: Longitudinal measurement of cortisol in association with mental health and experience of domestic violence and abuse: study protocol
Source: BMC Psychiatry. 2013 Jul 13;13:188. doi: 10.1186/1471-244X-13-188 (PMC3716999; doi:10.1186/1471-244X-13-188)
Supplement: Additional file 1 — Diurnal cortisol activity in women exposed to childhood abuse (CHA) and DVA. PDF table summarizing methodological information about publishes studies on HPA axis activity in association with exposure to violence and abuse. [file 1471-244X-13-188-S1.pdf]

Additional file 1 – Diurnal cortisol activity in women exposed to childhood abuse (CHA) and DVA

**Research (year), country**	**n =**	**Type of abuse**	**Cortisol protocol**	**Mental health measures**	**Findings**
Altemus et al (2003) [27]	31	Physical and sexual CHA	1 plasma sample + 6 saliva samples every 3 hours from 8:00 to 23:00	PTSD, MDD, anxiety	1) Salivary and plasma cortisol levels **did not differ** between PTSD patients and comparison subjects; 2) No difference in mean salivary or plasma cortisol in patients with PTSD and comorbid major depression and in those without depression
Seedat et al (2003), USA [28]	38	Physical and/or sexual IPV, CHA	1 morning plasma sample between 09:00-12:00	PTSD	1) Women with IPV trauma had significantly **lower** cortisol compared with controls; 2) Cortisol levels were not significantly correlated with PTSD symptom measures.

Research (year), country	n =	Type of abuse	Cortisol protocol	Mental health measures	Findings
Newport et al (2004), USA [29]	64	Repeated physical and sexual CHA	Plasma sample at 08:00	PTSD, MDD, anxiety	1) Baseline cortisol was **lower** in CHA survivors without MDD compared to non-abused controls.
Pico-Alfonso et al (2004), Spain [30]	162	Physical, sexual, psychological IPV, CHA	4 consecutive days 2 saliva samples at 08:00 and 20:00	PTSD, MDD, anxiety	1) IPV survivors had **higher** levels of evening cortisol; 2) IPV was the main factor predicting the alterations in cortisol levels; 3) Mental health status did not have a mediating effect on the impact of IPV on cortisol levels.
Griffin et al (2005), USA [31]	84	Physical, sexual, psychological IPV	1 morning plasma sample at 09:00-09:15	PTSD, MDD	1) Baseline cortisol levels in IPV survivors with PTSD and those with PTSD plus depression were significantly **lower** than the baseline cortisol levels of

Research (year), country	n =	Type of abuse	Cortisol protocol	Mental health measures	Findings
					the IPV survivors with no diagnosis and controls.
Inslicht et al (2006), USA [32]	49	Physical, sexual, emotional IPV	4 saliva samples at 1,4,9,11 h after awakening	PTSD, MDD	1) The PTSD group had **higher** levels of cortisol than IPV controls without PTSD; 2) More recent abuse was associated with higher cortisol; 3) Cortisol levels did not differ significantly between women with a sole diagnosis of PTSD and those who had PTSD and depression.
Johnson et al (2008), USA [33]	52	Physical, sexual, psychological IPV	4 saliva samples: awakening and 30-45-60 min post-awakening	PTSD, MDD	1) IPV-related PTSD severity is associated with a significantly **greater** cortisol output the first hour after awakening; 2) More chronic abuse is associated with lower CAR; 3) PTSD severity and

Research (year), country	n =	Type of abuse	Cortisol protocol	Mental health measures	Findings
					abuse chronicity did not significantly correlate; 4) There were no significant interactions between PTSD severity and abuse chronicity for CAR.
