# Supplementary material for: Longitudinal measurement of cortisol in association with mental health and experience of domestic violence and abuse: study protocol
Source: BMC Psychiatry. 2013 Jul 13;13:188. doi: 10.1186/1471-244X-13-188 (PMC3716999; doi:10.1186/1471-244X-13-188)
Supplement: Additional file 2 — Study plan and measurements. PDF table summarizing CEASE study plan and measurements. [file 1471-244X-13-188-S2.pdf]

**Additional file 2 - Study plan and measurements**

| Measurement | Instrument | Baseline | 3 month | 6 month |
|---|---|---|---|---|
| **HPA axis activity** | | | | |
| – Diurnal cortisol variation | Salivettes, Ultra performance liquid chromatography – tandem mass spectrometry (UPLC-MSMS) | • | • | • |
| – CAR | Salivettes, UPLC-MSMS | • | • | • |
| – Mean cortisol concentration | Salivettes, UPLC-MSMS | • | • | • |
| – Diurnal cortisol secretion covariates | Saliva collection diary | • | • | • |
| **Experience of violence and abuse** | | | | |

| Measurement | Instrument | Baseline | 3 month | 6 month |
|---|---|---|---|---|
| DVA | Composite Abuse Scale (CAS) | • | • | • |
| Childhood abuse | Childhood Trauma Questionnaire (CTQ) | • | | |
| **Mental health** | | | | |
| – Anxiety disorders | Generalized Anxiety Disorder (GAD-7) | • | • | • |
| – Depression | Patient Health Questionnaire Depression Scale (PHQ-9) | • | • | • |
| – Post Traumatic Stress Disorder (PTSD) | PTSD Symptom Scale: Self-Report Version (PSS-SR) | • | • | • |
| – Functional disability and impairment | Sheehan Disability Scale (SDS) | • | • | • |

| Measurement | Instrument | Baseline | 3 month | 6 month |
| --- | --- | --- | --- | --- |
| – Perception of stress | Perceived Stress Scale (PSS-10) | • | • | • |
| **Physical health** | | | | |
| – Abuse-related injuries and physical conditions | Miller Abuse Physical Symptom Injury Assessment Scale (MAPSAIS) | • | • | • |
| – Standing height | KaWe tape, Germany | • | | |
| – Weight | SECA scales, Germany | • | • | • |
| Socio-demographics | Socio-demographic questionnaire | • | | |
